# Supplementary material for: Neonatal and maternal outcomes following SARS-CoV-2 infection and COVID-19 vaccination: a population-based matched cohort study
Source: Nat Commun. 2023 Aug 29;14:5275. doi: 10.1038/s41467-023-40965-9 (PMC10465539; doi:10.1038/s41467-023-40965-9)
Supplement: Supplementary file 1 — Supplementary Information [file 41467_2023_40965_MOESM1_ESM.pdf]

SUPPLEMENTARY MATERIAL

Supplementary Table 1: Pre-matched characteristics of our exposed and unexposed pregnancies included in infection and vaccination analyses.

	Infected	Uninfected pre-matched controls	Vaccinated	Unvaccinated pre-matched controls
**Number of pregnancies**	4074	64559	11379	38354
**Median maternal age (min-max)**	29 (14-45)	30 (13-55)	32 (14-52)	30 (14-55)
**Maternal Deprivation**				
1 (most deprived)	1225 (30.1%)	15544 (24.1%)	1620 (14.2%)	9461 (24.7%)
2	1028 (25.2%)	13219 (20.5%)	1838 (16.2%)	7898 (20.6%)
3	681 (16.7 %)	11875 (18.4%)	2138 (18.8%)	7029 (18.3%)
4	675 (16.6%)	13337 (20.7%)	2864 (25.2%)	7896 (20.6%)
5 (least deprived)	465 (11.4%)	10530 (16.3%)	2,917 (25.6%)	6039 (15.7%)
Unknown/Missing	0 (0%)	54 (0.1%)	2 (<0.1%)	31 (0.1%)
**Maternal ethnicity**				
White	3611 (88.6%)	53045 (82.2%)	9845 (86.5%)	31947 (83.3%)
South Asian	183 (4.5%)	2210 (3.4%)	433 (3.8%)	1318 (3.4%)
Black/Caribbean/African	57 (1.4%)	1148 (1.8%)	132 (1.2%)	743 (1.9%)
Other/mixed ethnicity	156 (3.8%)	2427 (3.8%)	468 (4.1%)	1481 (3.9%)
Unknown/Missing	67 (1.6%)	5729 (8.9%)	501 (4.4%)	2865 (7.5%)
**Maternal urban/rural status**				
Large urban areas	1616 (39.7%)	22940 (35.5%)	4369 (38.4%)	13471 (35.1%)
Other urban areas	1644 (40.4%)	23903 (37.0%)	3548 (31.2%)	14215 (37.1%)
Accessible small towns	248 (6.1%)	5202 (8.1%)	961 (8.4%)	3118 (8.1%)
Remote small towns	89 (2.2%)	2089 (3.2%)	346 (3.0%)	1213 (3.2%)
Accessible rural areas	373 (9.2%)	7140 (11.1%)	1459 (12.8%)	4259 (11.1%)
Remote rural areas	81 (2.0%)	2899 (4.5%)	601 (5.3%)	1805 (4.7%)
Unknown/Missing	23 (0.6%)	386 (0.6%)	95 (0.8%)	273 (0.7%)
**Parity**				
0	1655 (40.6%)	27154 (42.1%)	4824 (42.4%)	15940 (41.6%)
1+	2298 (56.4%)	35456 (54.9%)	6164 (54.2%)	21219 (55.3%)
Unknown/missing	121 (3.0%)	1949 (3.0%)	391 (3.4%)	1195 (3.1%)
**Maternal clinical vulnerability**				
Not clinically vulnerable	3012 (73.9%)	47373 (73.4%)	8411 (73.9%)	28301 (73.8%)
Clinically vulnerable	1035 (25.4%)	16595 (25.7%)	2839 (24.9%)	9749 (25.4%)
Extremely vulnerable	27 (0.7%)	591 (0.9%)	129 (1.1%)	304 (0.8%)
**Maternal diabetes**				
No diabetes	3749 (92.0%)	59959 (92.9%)	10411 (91.5%)	35383 (92.3%)
Pre-existing diabetes	28 (0.7%)	569 (0.9%)	147 (1.3%)	313 (0.8%)
Gestational diabetes	297 (7.3%)	4031 (6.2%)	821 (7.2%)	2658 (6.9%)
**Maternal smoking status**				
Non-smoker	2785 (68.4%)	42699 (66.1%)	8612 (75.7%)	25390 (66.2%)
Ex-smoker	819 (20.1%)	12981 (20.1%)	2074 (18.2%)	7663 (20.0%)
Smoker	468 (11.5%)	8573 (13.3%)	679 (6.0%)	5188 (13.5%)
Unknown/Missing	2 (<0.1%)	306 (0.5%)	14 (0.1%)	113 (0.3%)
**Body mass index**				
Underweight	90 (2.2%)	1759 (2.7%)	176 (1.5%)	1022 (2.7%)
Healthy weight	1411 (34.6%)	24253 (37.6%)	4324 (38%)	14299 (37.3%)
Overweight	1288 (31.6%)	19232 (29.8%)	3431 (30.2%)	11504 (30.0%)
Obese/severely obese	1203 (29.5%)	17585 (27.2%)	3183 (28%)	10577 (27.6%)
Unknown/Missing	82 (2.0%)	1730 (2.7%)	265 (2.3%)	952 (2.5%)

The data presented relates to Cohort 1: singleton pregnancies ending in a live or stillbirth at ≥20+0 gestation used for analyses of stillbirth and extended perinatal death outcomes.

Supplementary Table 2: Lag in days between infection (or matching) and end of pregnancy for pregnancies ending in preterm (<37+0 gestation) and very preterm births (<32+0 gestation).

Lag from exposure (infection or, for controls, gestation at matching with infected case)	Exposure group	Spontaneous preterm births	Provider-initiated preterm births	Total preterm births (includes preterm births missing delivery onset)
**Preterm births**				
0-6 days	Infected	17 (11.4%)	18 (15.1%)	35 (12.8%)
	Uninfected	16 (4.4%)	20 (8.1%)	36 (5.9%)
7-27 days	Infected	34 (22.8%)	30 (25.2%)	65 (23.7%)
	Uninfected	68 (18.8%)	42 (17.1%)	111 (18.2%)
>=28 days	Infected	98 (65.8%)	71 (59.7%)	174 (63.5%)
	Uninfected	277 (76.7%)	184 (74.8%)	464 (75.9%)
**Very preterm births**				
0-6 days	Infected	2 (9.1%)	3 (15.0%)	5 (13.2%)
	Uninfected	2 (3.9%)	0	2 (2.9%)
7-27 days	Infected	5 (22.7%)	11 (73.3%)	16 (42.1%)
	Uninfected	12 (23.5%)	2 (12.5%)	14 (20.6%)
>=28 days	Infected	15 (68.2%)	1 (6.7%)	17 (44.7%)
	Uninfected	37 (72.5%)	14 (87.5%)	52 (76.5%)

Supplementary Table 3: Sub-types of provider-initiated preterm births for Cohort 5.

	Infected	Uninfected controls	Vaccinated	Unvaccinated controls
Provider-initiated preterm birth	119	246	233	464
Provider-initiated preterm birth with induction of labour (any subsequent mode of delivery)	31	90	54	132
Provider-initiated preterm birth with pre-labour caesarean section	76	151	177	324
Provider-initiated preterm birth type unknown (delivery during critical care admission)	12	5	2	8

Supplementary Table 4: Comparison between odds ratios (calculated using conditional logistic regression) and risk ratios 9calculated using conditional Poisson regression) for the association between exposure to SARS-CoV-2 infection during pregnancy and neonatal and maternal outcomes.

Cohort (Outcome)	Infection status	Odds ratio accounting for only matching* [95% CI]	P value*	Adjusted odds ratio** [95% CI]	P value**	Risk ratio accounting for only matching* [95% CI]	P value*	Adjusted risk ratio** [95% CI]	P value**
**Neonatal**									
1 (Stillbirths)	Infected	1.02 [0.55-1.88]	0.94	1.08 [0.56-2.05]	0.82	1.02 [0.54-1.83]	0.94	1.08 [0.55-2.01]	0.81
	Uninfected	Ref	-	Ref	-	Ref	-	Ref	-
1 (Extended perinatal death)	Infected	1.13 [0.70-1.82]	0.62	1.13 [0.68-1.89]	0.63	1.13 [0.69-1.80]	0.62	1.13 [0.67-1.86]	0.63
	Uninfected	Ref	-	Ref		Ref	-	Ref	-
2 (Neonatal death)	Infected	2.25 [0.95-5.34]	0.07	-	-	2.25 [0.92-5.32]	0.07	-	-
	Uninfected	Ref	-	-	-	Ref	-	-	-
3 (Small for gestational age (<10th percentile)***)	Infected	0.91 [0.77-1.08]	0.26	1.04 [0.86-1.26]	0.67	0.92 [0.77-1.08]	0.29	1.05 [0.87-1.25]	0.63
	Uninfected	Ref	-	Ref	-	Ref	-	Ref	-
3 (Very small for gestational age (<3rd percentile)***)	Infected	0.88 [0.61-1.26]	0.48	1.02 [0.65-1.61]	0.93	0.88 [0.60-1.25]	0.48	1.02 [0.64-1.61]	0.92
	Uninfected	Ref	-	Ref	-	Ref	-	Ref	-
4 (Low Apgar score (<7)***)	Infected	0.99 [0.74-1.32]	0.95	0.96 [0.71-1.30]	0.80	0.99 [0.74-1.31]	0.95	0.96 [0.71-1.29]	0.81
	Uninfected	Ref	-	Ref	-	Ref	-	Ref	-
4 (Very low Apgar score (<4)***)	Infected	0.77 [0.38-1.54]	0.46	0.87 [0.42-1.81]	0.72	0.77 [0.36-1.48]	0.46	0.87 [0.40-1.76]	0.72
	Uninfected	Ref	-	Ref	-	Ref	-	Ref	-
5 (Preterm birth)	Infected	1.37 [1.18-1.59]	<0.001	1.36 [1.16-1.59]	<0.001	1.35 [1.17-1.55]	<0.001	1.33 [1.14-1.54]	<0.001
	Uninfected	Ref	-	Ref	-	Ref	-	Ref	-
5 (Spontaneous preterm birth***)	Infected	1.25 [1.03-1.52]	0.03	1.27 [1.03-1.56]	0.03	1.24 [1.02-1.50]	0.03	1.24 [1.01-1.51]	0.04
	Uninfected	Ref	-	Ref	-	Ref	-	Ref	-
5 (Provider-initiated preterm birth***)	Infected	1.46 [1.17-1.82]	<0.001	1.42 [1.11-1.81]	0.005	1.45 [1.16-1.80]	<0.001	1.43 [1.12-1.81]	0.004
	Uninfected	Ref	-	Ref	-	Ref	-	Ref	-
6 (Very preterm birth)	Infected	1.68 [1.13-2.49]	0.01	1.90 [1.20-3.02]	0.01	1.68 [1.12-2.48]	0.01	1.90 [1.19-3.01]	0.006

	Uninfected	Ref	-	Ref	-	Ref	-	Ref	-
6 (Spontaneous very preterm birth***)	Infected	1.29 [0.77-2.13]	0.31	1.31 [0.78-2.18]	0.31	1.29 [0.77-2.10]	0.31	1.31 [0.77-2.16]	0.31
	Uninfected	Ref	-	Ref	-	Ref	-	Ref	-
6 (Provider-initiated very preterm birth***)	Infected	2.81 [1.39-5.69]	<0.001	2.63 [1.23-5.62]	0.01	2.81 [1.38-5.72]	0.004	2.63 [1.22-5.68]	0.01
	Uninfected	Ref	-	Ref	-	Ref	-	Ref	-
**Maternal**									
7 (Critical care and/or Death)	Infected	1.83 [1.51-2.21]	<0.001	1.72 [1.39-2.12]	<0.001	1.80 [1.49-2.17]	<0.001	1.69 [1.38-2.08]	<0.001
	Uninfected	Ref	-	Ref	-	Ref	-	Ref	-
8 (Venous thromboembolism)	Infected	2.72 [1.65-4.49]	<0.001	2.53 [1.47-4.35]	<0.001	2.72 [1.64-4.50]	<0.001	2.53 [1.47-4.36]	<0.001
	Uninfected	Ref	-	Ref	-	Ref	-	Ref	-
9 (Hypertensive disorders of pregnancy)	Infected	0.85 [0.72-1.01]	0.06	0.82 [0.68-0.98]	0.03	0.86 [0.72-1.01]	0.07	0.82 [0.69-0.98]	0.03
	Uninfected	Ref	-	Ref	-	Ref	-	Ref	-
10 (Pregnancy-related bleeding)	Infected	0.96 [0.90-1.05]	0.33	0.97 [0.90-1.04]	0.39	0.98 [0.93-1.03]	0.46	0.98 [0.93-1.04]	0.47
	Uninfected	Ref	-	Ref	-	Ref	-	Ref	-

CI=Confidence Interval

*Matched for maternal age, gestation at infection/matching and seasons of conception and additionally for health board of residence (Greater Glasgow & Clyde or Lanarkshire versus all other health boards) for hypertensive disorders of pregnancy and pregnancy-related bleeding.

**Adjustment for deprivation only for analyses with the following outcomes: spontaneous very preterm birth and provider-initiated very preterm birth. Adjustment for parity and deprivation only for analyses with the following outcomes: stillbirth, extended perinatal death, very low Apgar score and venous thromboembolism. Adjustment for all covariates apart from parity for the following outcomes: preterm birth, spontaneous preterm birth, provider-initiated preterm birth and very preterm birth. Adjustment for all covariates for analyses for the following outcomes: small for gestational age, very small for gestational age, low Apgar score, maternal critical care or death, hypertensive disorders of pregnancy and pregnancy-related bleeding.

***For these outcomes there is not an exact match of three uninfected controls to each neonate exposed to infection as neonates with missing outcome data have been removed from analysis; see Supplementary Table 11 for further details on levels of missing data.

Supplementary Table 5: Association between exposure to SARS-CoV-2 during pregnancy at 6 weeks preconception to ≤19+6 weeks gestation and neonatal and maternal outcomes (reinfections spanning both gestational periods excluded from subgroup analysis), calculated using conditional logistic regression.

Cohort	Infection Status	Number of pregnancies/ neonates	Number with outcome	% s with outcome	Odds ratio accounting for only matching [95% CI]*	P value*	Adjusted odds ratio** [95% CI]	P value**
**Neonatal**								
1 (Stillbirths)	Infected	1269	1	0.08%	0.16 [0.02-1.18]	0.07	-	-
	Uninfected	3807	19	0.5%	Ref		-	-
1 (Extended perinatal death)	Infected	1269	4	0.3%	0.44 [0.16-1.27]	0.13	0.33 [0.10-1.01]	0.05
	Uninfected	3807	27	0.7%	Ref	-	Ref	-
2 (Neonatal death)	Infected	1268	3	0.2%	1.80 [0.43-7.53]	0.42	-	-
	Uninfected	3804	5	0.1%	Ref	-	-	-
3 (Small for gestational age (<10th percentile)***)	Infected	1247	58	4.7%	0.87 [0.64-1.17]	0.36	1.00 [0.70-1.41]	0.98
	Uninfected	3644	195	5.4%	Ref	-	Ref	-
3 (Very small for gestational age (<3rd percentile)***)	Infected	1247	14	1.1%	0.98 [0.53-1.79]	0.94	0.89 [0.47-1.69]	0.72
	Uninfected	3644	42	1.2%	Ref	-	Ref	-
4 (Low Apgar score (<7)***)	Infected	1156	20	1.8%	0.82 [0.49-1.35]	0.43	0.76 [0.44-1.32]	0.34
	Uninfected	3342	70	2.0%	Ref	-	Ref	-
4 (Very low Apgar score (<3)***)	Infected	1180	5	0.4%	0.83 [0.31-2.26]	0.72	-	-
	Uninfected	3540	17	0.5%	Ref	-	-	-
5 (Preterm birth)	Infected	1290	88	6.8%	1.21 [0.94-1.56]	0.15	1.15 [0.86-1.53]	0.34
	Uninfected	3870	221	5.7%	Ref	-	Ref	-
5 (Spontaneous preterm birth***)	Infected	1287	47	3.7%	0.99 [0.70-1.38]	0.93	0.92 [0.63-1.34]	0.67
	Uninfected	3858	143	3.7%	Ref	-	Ref	-
5 (Provider-initiated preterm birth***)	Infected	1287	38	3.0%	1.52 [1.03-2.26]	0.04	1.51 [0.96-2.37]	0.07
	Uninfected	3858	75	1.9%	Ref	-	Ref	-
6 (Very preterm birth)	Infected	1349	13	1.0%	1.03 [0.55-1.93]	0.94	0.94 [0.49-1.80]	0.84
	Uninfected	4047	38	0.9%	Ref	-	Ref	-
6 (Spontaneous very preterm birth***)	Infected	1348	11	0.8%	1.18 [0.59-2.37]	0.64	1.13 [0.55-2.33]	0.73
	Uninfected	4043	28	0.7%	Ref	-	Ref	-
6 (Provider-initiated very preterm birth***)	Infected	1348	1	0.1%	0.33 [0.04-2.63]	0.30	-	-
	Uninfected	4043	9	0.2%	Ref	-	-	-
**Maternal**								
7 (Critical care and/or death)	Infected	1269	24	2.0%	0.87 [0.54-1.37]	0.54	0.85 [0.51-1.42]	0.54
	Uninfected	3807	83	2.2%	Ref	-	Ref	-
8 (Venous thromboembolism)	Infected	1269	6	0.5%	1.50 [0.56-4.00]	0.42	-	-
	Uninfected	3807	12	0.3%	Ref	-	-	-
9 (Hypertensive Disorders of Pregnancy**)	Infected	1268	66	5.2%	1.02 [0.77-1.36]	0.88	1.02 [0.75-1.38]	0.92
	Uninfected	3804	194	5.1%	Ref	-	Ref	-
10 (Pregnancy-related bleeding**)	Infected	1263	566	44.8%	1.00 [0.88-1.13]	0.96	0.98 [0.86-1.12]	0.77
	Uninfected	3789	1701	44.9%	Ref	-	Ref	-

CI=Confidence Interval

*Matched for maternal age, gestation at infection/matching and seasons of conception and additionally for health board (Glasgow or Lanarkshire versus all other health boards) for hypertensive disorders of pregnancy and pregnancy-related bleeding.

**Adjustment for deprivation only for analyses with the following outcomes: very preterm birth, spontaneous very preterm birth and provider-initiated very preterm birth. Adjustment for parity and deprivation only for analyses with the following outcomes: extended perinatal death, very small for gestational age and low Apgar score. Adjustment for all covariates for analyses apart from parity for preterm birth, spontaneous preterm birth and provider-initiated preterm birth. Adjustment for all covariates for analyses for the following outcomes: small for gestational age, very small for gestational age, low Apgar score, maternal critical care and/or death, hypertensive disorders of pregnancy and pregnancy-related bleeding.
***For these outcomes there is not an exact match of three uninfected controls to each neonate exposed to infection as babies with missing outcome data have been removed from analysis; see Supplementary Table 11 for further details on levels of missing data.

Supplementary Table 6: Association between exposure to SARS-CoV-2 during pregnancy at ≥20 weeks gestation and neonatal and maternal outcomes (reinfections spanning both gestational periods excluded from subgroup analysis), calculated using conditional logistic regression.

Cohort	Infection status	Number of neonates	Number with outcome	% with outcome	Odds ratio accounting for only matching* [95% CI]	P value*	Adjusted odds ratio** [95% CI]	P value**
**Neonatal**								
1 (Stillbirths)	Infected	2774	13	0.5%	1.86 [0.93-3.71]	0.08	2.01 [0.92-4.39]	0.08
	Uninfected	8322	21	0.3%	Ref	-	Ref	-
1 (Extended perinatal death)	Infected	2774	19	0.7%	1.73 [0.98-3.04]	0.06	1.77 [0.95-3.31]	0.07
	Uninfected	8322	33	0.4%	Ref	-	Ref	-
2 (Neonatal death)	Infected	2761	6	0.2%	2.57 [0.86-7.65]	0.09	-	-
	Uninfected	8283	7	0.1%	Ref	-	-	-
3 (Small for gestational age (<10th percentile)***)	Infected	2758	127	4.6%	0.95 [0.77-1.16]	0.61	1.10 [0.87-1.38]	0.43
	Uninfected	8274	410	5.0%	Ref	-	Ref	-
3 (Very small for gestational age (<3rd percentile)***)	Infected	2758	24	0.9%	0.84 [0.53-1.32]	0.45	1.05 [0.56-1.92]	0.87
	Uninfected	8274	89	1.1%	Ref	-	Ref	-
4 (Low Apgar score (<7)***)	Infected	2576	44	1.7%	1.10 [0.77-1.56]	0.61	1.05 [0.72-1.54]	0.80
	Uninfected	7728	117	1.5%	Ref	-	Ref	-
4 (Very low Apgar score (<3)***)	Infected	2576	5	0.2%	0.71 [0.27-1.90]	0.50	-	-
	Uninfected	7728	20	0.3%	Ref	-	-	-
5 (Preterm birth)	Infected	2325	188	8.1%	1.46 [1.22-1.75]	<0.001	1.46 [1.20-1.77]	<0.001
	Uninfected	6975	394	5.7%	Ref	-	Ref	-
5 (Spontaneous preterm birth***)	Infected	2322	105	4.5%	1.46 [1.15-1.86]	0.002	1.50 [1.16-1.94]	0.002
	Uninfected	6964	219	3.1%	Ref	-	Ref	-
5 (Provider-initiated preterm birth***)	Infected	2322	80	3.4%	1.40 [1.07-1.83]	0.01	1.37 [1.01-1.84]	0.04
	Uninfected	6964	173	2.5%	Ref	-	Ref	-
6 (Very preterm birth)	Infected	1486	25	1.7%	2.50 [1.47-4.25]	<0.001	2.59 [1.47-4.56]	<0.001
	Uninfected	4458	30	0.7%	Ref	-	Ref	-
6 (Spontaneous very preterm birth***)	Infected	1486	11	0.7%	1.44 [0.70-2.94]	0.33	1.51 [0.71-3.22]	0.28
	Uninfected	4458	23	0.5%	Ref	-	Ref	-
6 (Provider-initiated very preterm birth***)	Infected	1486	14	0.9%	6.00 [2.42-14.87]	<0.001	-	-
	Uninfected	4458	7	0.2%	Ref	-	-	-
**Maternal**								
7 (Critical care and/or death)	Infected	2774	150	5.4%	2.26 [1.82-2.80]	<0.001	2.12 [1.67-2.69]	<0.001
	Uninfected	8322	204	2.5%	Ref	-	Ref	-
8 (Venous thromboembolism)	Infected	2772	23	0.8%	3.45 [1.90-6.28]	<0.001	2.94 [1.53-5.63]	0.001
	Uninfected	8316	20	0.2%	Ref	-	Ref	-
9 (Hypertensive Disorders of Pregnancy**)	Infected	2772	110	4.0%	0.77 [0.62-0.95]	0.02	0.73 [0.58-0.92]	0.01
	Uninfected	8316	423	5.1%	Ref	-	Ref	-
10 (Pregnancy-related bleeding**)	Infected	2679	1085	40.5%	0.95 [0.87-1.03]	0.22	0.96 [0.88-1.06]	0.44

	Uninfected	8037	3365	41.9%	Ref	-	Ref	-

CI=Confidence Interval

*Matched for maternal age, gestation at infection/matching and seasons of conception and additionally for health board (Glasgow or Lanarkshire versus all other health boards) for hypertensive disorders of pregnancy and pregnancy-related bleeding.

**Adjustment for deprivation only for analyses with the following outcomes: very preterm birth, spontaneous very preterm birth and provider-initiated very preterm birth. Adjustment for parity and deprivation only for analyses with the following outcomes: stillbirth, extended perinatal death and venous thromboembolism. Adjustment for all covariates for analyses apart from parity for preterm birth, spontaneous preterm birth and provider-initiated preterm birth. Adjustment for all covariates for analyses for the following outcomes: small for gestational age, very small for gestational age, low Apgar score maternal critical care and/or death, hypertensive disorders of pregnancy and pregnancy-related bleeding.

***For these outcomes there is not an exact match of three uninfected controls to each neonate exposed to infection as neonates with missing outcome data have been removed from analysis; see Supplementary Table 11 for further details on levels of missing data.

Supplementary Table 7: Association between SARS-CoV-2 infection and hypertension/bleeding outcomes, stratified by health board of residence, calculated using conditional logistic regression.

Outcome	Health Board of residence	Infection Status	Number of neonates	Number with outcome	% with outcome	Odds ratio accounting for only matching* [95% CI]	P value*	Adjusted odds ratio** [95% CI]	P value**
Hypertensive Disorders of Pregnancy	NHS Greater Glasgow & Clyde/NHS Lanarkshire	Infected	1885	83	4.4%	0.93 [0.73-1.21]	0.61	0.87 [0.66-1.14]	0.31
		Uninfected	5655	265	4.7%	Ref	-	Ref	-
	All other health boards	Infected	2186	95	4.3%	0.78 [0.62-0.99]	<0.05	0.80 [0.62-1.02]	0.07
		Uninfected	6558	359	5.5%	Ref	-	Ref	-
Pregnancy-related bleeding	NHS Greater Glasgow & Clyde/NHS Lanarkshire	Infected	1823	823	45.1%	0.95 [0.85-1.05]	0.32	0.93 [0.83-1.03]	0.16
		Uninfected	5469	2543	46.5%	Ref	-	Ref	-
	All other health boards	Infected	2150	842	39.2%	0.98 [0.89-1.08]	0.69	1.01 [0.91-1.12]	0.88
		Uninfected	6450	2558	39.7%	Ref	-	Ref	-

CI=Confidence Interval
*Matched for maternal age, gestation at infection/matching and seasons of conception.
**Adjustment for all covariates for both maternal outcomes.

Supplementary Table 8: Association between vaccination and hypertension/bleeding outcomes stratified by health board, calculated using conditional logistic regression.

**Outcome**	**Health board**	**Vaccination Status**	**Number of neonates**	**Number with outcome**	**% with outcome**	**Odds ratio accounting for only matching* [95% CI]**	**P value***	**Adjusted odds ratio** [95% CI]**	**P value****
Hypertensive Disorders of Pregnancy	NHS Greater Glasgow & Clyde/NHS Lanarkshire	Vaccinated	3620	178	4.9%	1.01 [0.84-1.22]	0.92	0.89 [0.72-1.09]	0.27
		Unvaccinated	7240	353	4.9%	Ref	-	Ref	-
	All other health boards	Vaccinated	7753	460	5.9%	0.99 [0.88-1.11]	0.83	0.89 [0.79-1.01]	0.06
		Unvaccinated	15506	931	6.0%	Ref	-	Ref	-
Pregnancy-related bleeding	NHS Greater Glasgow & Clyde/NHS Lanarkshire	Vaccinated	3557	1771	49.7%	1.06 [0.98-1.15]	0.14	0.99 [0.91-1.08]	0.89
		Unvaccinated	7114	3436	48.3%	Ref	-	Ref	-
	All other health boards	Vaccinated	7673	3424	44.6%	1.11 [1.05-1.17]	<.001	1.06 [1.00-1.12]	0.06
		Unvaccinated	15346	6464	42.1%	Ref	-	Ref	-

*Matched for maternal age, gestation at infection/matching and seasons of conception.
**Adjustment for all covariates for both maternal outcomes.

Supplementary Table 9: Comparison between odds ratios (calculated using conditional logistic regression) and risk ratios (calculated using conditional Poisson regression) for the association between exposure to COVID-19 vaccination during pregnancy and neonatal and maternal outcomes.

Cohort (Outcome)	Vaccination status	Odds ratio accounting for only matching* [95% CI]	P value*	Adjusted odds ratio** [95% CI]	P value**	Risk ratio accounting for only matching* [95% CI]	P value*	Adjusted risk ratio** [95% CI]	P value**
**Neonatal**									
1 (Stillbirths)	Vaccinated	1.08 [0.68-1.70]	0.75	1.03 [0.63-1.68]	0.91	1.07 [0.68-1.67]	0.76	1.04 [0.64-1.68]	0.86
	Unvaccinated	Ref	-	Ref	-	Ref	-	Ref	-
1 (Extended perinatal death)	Vaccinated	0.92 [0.63-1.34]	0.66	0.97 [0.64-1.48]	0.90	0.92 [0.63-1.32]	0.66	0.98 [0.64-1.48]	0.93
	Unvaccinated	Ref	-	Ref	-	Ref	-	Ref	-
2 (Neonatal death)	Vaccinated	0.56 [0.29-1.06]	0.07	0.51 [0.26-1.01]	0.05	0.56 [0.28-1.02]	0.07	0.51 [0.25-0.98]	0.05
	Unvaccinated	Ref	-	Ref	-	Ref	-	-	-
3 (Small for gestational age (<10th percentile)***)	Vaccinated	0.89 [0.79-0.99]	0.04	1.02 [0.90-1.16]	0.79	0.89 [0.80-1.00]	0.04	1.01 [0.90-1.15]	0.81
	Unvaccinated	Ref	-	Ref	-	Ref	-	Ref	-
3 (Very small for gestational age (<3rd percentile)***)	Vaccinated	0.90 [0.71-1.14]	0.40	1.06 [0.81-1.40]	0.66	0.90 [0.71-1.14]	0.40	1.07 [0.81-1.40]	0.63
	Unvaccinated	Ref	-	Ref	-	Ref	-	Ref	-
4 (Low Apgar score (<7)***)	Vaccinated	1.08 [0.90-1.30]	0.41	1.13 [0.93-1.37]	0.23	1.08 [0.90-1.30]	0.41	1.13 [0.92-1.37]	0.24
	Unvaccinated	Ref	-	Ref	-	Ref	-	Ref	-
4 (Very low Apgar score (<4)***)	Vaccinated	1.11 [0.68-1.80]	0.67	1.32 [0.79-2.23]	0.29	1.11 [0.67-1.79]	0.67	1.33 [0.78-2.23]	0.29
	Unvaccinated	Ref	-	Ref	-	Ref	-	Ref	-
5 (Preterm birth)	Vaccinated	0.93 [0.84-1.03]	0.15	0.96 [0.86-1.06]	0.41	0.93 [0.85-1.03]	0.16	0.95 [0.86-1.06]	0.38
	Unvaccinated	Ref	-	Ref	-	Ref	-	Ref	-
5 (Spontaneous preterm birth***)	Vaccinated	0.89 [0.79-1.01]	0.08	0.95 [0.83-1.08]	0.43	0.90 [0.79-1.01]	0.09	0.95 [0.83-1.08]	0.42
	Unvaccinated	Ref	-	Ref	-	Ref	-	Ref	-
5 (Provider-initiated preterm birth***)	Vaccinated	1.00 [0.86-1.18]	0.97	0.95 [0.79-1.13]	0.55	1.00 [0.86-1.17]	0.97	0.94 [0.79-1.12]	0.51
	Unvaccinated	Ref	-	Ref	-	Ref	-	Ref	-
6 (Very preterm birth)	Vaccinated	0.88 [0.67-1.16]	0.36	0.89 [0.64-1.23]	0.47	0.88 [0.67-1.15]	0.36	0.89 [0.64-1.22]	0.47

	Unvaccinated	Ref	-	Ref	-	Ref	-	Ref	-
6 (Spontaneous very preterm birth***)	Vaccinated	0.93 [0.68-1.28]	0.67	0.92 [0.63-1.35]	0.67	0.93 [0.68-1.27]	0.67	0.92 [0.62-1.35]	0.68
	Unvaccinated	Ref	-	Ref	-	Ref	-	Ref	-
6 (Provider-initiated very preterm birth***)	Vaccinated	0.58 [0.31-1.07]	0.08	0.69 [0.35-1.28]	0.22	0.58 [0.30-1.04]	0.08	0.67 [0.34-1.25]	0.22
	Unvaccinated	Ref	-	Ref	-	Ref	-	Ref	-
**Maternal**									
7 (Critical care and/or Death)	Vaccinated	0.92 [0.79-1.07]	0.26	0.86 [0.73-1.01]	0.07	0.92 [0.79-1.06]	0.26	0.86 [0.73-1.01]	0.07
	Unvaccinated	Ref	-	Ref	-	Ref	-	Ref	-
8 (Venous thromboembolism)	Vaccinated	0.60 [0.35-1.04]	0.07	0.62 [0.35-1.09]	0.10	0.61 [0.34-1.02]	0.07	0.63 [0.35-1.07]	0.10
	Unvaccinated	Ref	-	Ref	-	Ref	-	Ref	-
9 (Hypertensive disorders of pregnancy)	Vaccinated	0.99 [0.90-1.10]	0.89	0.89 [0.80-0.99]	0.04	0.99 [0.90-1.09]	0.90	0.90 [0.82-1.00]	0.05
	Unvaccinated	Ref	-	Ref	-	Ref	-	Ref	-
10 (Pregnancy-related bleeding)	Vaccinated	1.09 [1.04-1.14]	<0.001	1.04 [0.99-1.09]	0.14	1.05 [1.01-1.09]	0.005	1.02 [0.99-1.06]	0.25
	Unvaccinated	Ref	-	Ref	-	Ref	-	Ref	-

CI=Confidence Interval

*Matched for maternal age and gestation at vaccination/matching and additionally for health board of residence (Greater Glasgow & Clyde or Lanarkshire versus all other health boards) for hypertensive disorders of pregnancy and pregnancy-related bleeding.

**Adjustment for deprivation only for analyses with the following outcomes: provider-initiated very preterm birth. Adjustment for parity and deprivation only for analyses with the following outcomes: stillbirth, neonatal death, very low Apgar score and venous thromboembolism. Adjustment for all covariates apart from parity for the following outcomes: preterm birth, spontaneous preterm birth, provider-initiated preterm birth, very preterm birth, and spontaneous very preterm birth. Adjustment for all covariates for analyses for the following outcomes: extended perinatal death, small for gestational age, very small for gestational age, low Apgar score, maternal critical care or death, hypertensive disorders of pregnancy and pregnancy-related bleeding.

***For these outcomes there is not an exact match of two unvaccinated controls to each neonate exposed to vaccination as neonates with missing outcome data have been removed from analysis; see Supplementary Table 11 for further details on levels of missing data.

Supplementary Table 10: ICD-10 codes and critical care admission codes (drawn from the Scottish Intensive Care Society Audit Group [SICSAG] dataset) used to identify hypertensive disorders of pregnancy, pregnancy-related bleeding, and venous thromboembolism.

Outcome		Condition
**Hypertensive disorders of pregnancy**	**ICD-10**	
	O11	Pre-eclampsia superimposed on chronic hypertension
	O13	Gestational [pregnancy-induced] hypertension
	O14.0	Mild to moderate pre-eclampsia
	O14.1	Severe pre-eclampsia
	O14.2	HELLP syndrome
	O14.9	Pre-eclampsia, unspecified
	O15.0	Eclampsia in pregnancy
	O15.1	Eclampsia in labour
	O15.2	Eclampsia in the puerperium
	O15.9	Eclampsia, unspecified as to time period
	O16	Unspecified maternal hypertension
	**SICSAG**	
	217	Pregnancy induced hypertension
	215	Pre-eclampsia
	216	Pre-eclampsia/eclampsia
	99	HELLP syndrome
	74	Eclampsia
	107	Hypertension
	167	Other hypertension
**Pregnancy-related bleeding**		***Obstetric haemorrhage***
	**ICD-10**	
	O44.1	Placenta praevia with haemorrhage
	O45.0	Premature separation of placenta with coagulation defect
	O45.8	Other premature separation of placenta
	O45.9	Premature separation of placenta, unspecified
	O46.0	Antepartum haemorrhage with coagulation defect
	O46.8	Other antepartum haemorrhage
	O46.9	Antepartum haemorrhage, unspecified
	O67.0	Intrapartum haemorrhage with coagulation defect
	O67.8	Other intrapartum haemorrhage
	O67.9	Intrapartum haemorrhage, unspecified
	O69.4	Labour and delivery complicated by haemorrhage from vasa praevia
	O72.0	Third stage haemorrhage
	O72.1	Other immediate postpartum haemorrhage
	O72.2	Delayed and secondary postpartum haemorrhage
	O72.3	Postpartum coagulation defects
	**SICSAG**	
	20	Antepartum haemorrhage
	199	Peripartum haemorrhage
	214	Postpartum haemorrhage
	114	Hysterectomy
	113	Hypovolaemic/haemorrhagic shock
	33	Bleeding-laceration/tear
		Early pregnancy bleeding
	**ICD-10**	
	O03.1	Incomplete spontaneous abortion (miscarriage), complicated by haemorrhage

	O03.6	Complete spontaneous abortion (miscarriage), complicated by haemorrhage
	O04.1	Incomplete medical abortion (TOP), complicated by haemorrhage
	O04.6	Complete medical abortion (TOP), complicated by haemorrhage
	O05.1	Incomplete other abortion, complicated by haemorrhage
	O05.6	Complete other abortion, complicated by haemorrhage
	O06.1	Incomplete unspecified abortion, complicated by haemorrhage
	O06.6	Complete unspecified abortion, complicated by haemorrhage
	O07.1	Failed medical abortion, complicated by haemorrhage
	O07.6	Other and unspecified failed medical abortion, complicated by haemorrhage
	O08.1	Delayed or excessive haemorrhage following abortion and ectopic and molar pregnancy
	O20.0	Threatened abortion
	O20.8	Other haemorrhage in early pregnancy
	O20.9	Haemorrhage in early pregnancy, unspecified
	**SICSAG**	
	75	Ectopic pregnancy
		Disseminated intravascular coagulation
	**ICD-10**	
	D65	Disseminated intravascular coagulation
	**SICSAG**	
	70	Disseminated intravascular coagulation
**Venous thromboembolism**	**ICD-10**	
	I26.0	Pulmonary embolism with mention of acute cor pulmonale
	I26.9	Pulmonary embolism without mention of acute cor pulmonale
	I80.1	Phlebitis and thrombophlebitis of femoral vein
	I80.2	Phlebitis and thrombophlebitis of other deep vessels of lower extremities
	I80.3	Phlebitis and thrombophlebitis of lower extremities, unspecified
	O08.2	Embolism following abortion and ectopic and molar pregnancy
	O22.3	Deep thrombophlebitis in pregnancy
	O87.1	Deep phlebothrombosis in the puerperium
	O88.2	Obstetric blood-clot embolism
	I80.8	Phlebitis and thrombophlebitis of other sites
	I80.9	Phlebitis and thrombophlebitis of unspecified site
	I81	Portal vein thrombosis
	I82.0	Budd Chiari syndrome
	I82.1	Thrombophlebitis migrans
	I82.2	Embolism and thrombosis of vena cava
	I82.3	Embolism and thrombosis of renal vein
	I82.8	Embolism and thrombosis of other specified veins
	I82.9	Embolism and thrombosis of unspecified vein
	O22.9	Venous complication in pregnancy, unspecified
	O87.9	Venous complication in the puerperium, unspecified
	G08	Intracranial and intraspinal phlebitis and thrombophlebitis
	I63.6	Cerebral infarction due to cerebral venous thrombosis, nonpyogenic
	I67.6	Nonpyogenic thrombosis of intracranial venous system
	O22.5	Cerebral venous thrombosis in pregnancy
	O87.3	Cerebral venous thrombosis in the puerperium
	**SICSAG**	
	219	Pulmonar embolus
	220	Pulmonary embolism
	221	Pulmonary embolus
	225	Pulmonary thromboembolism
	275	Venous thrombosis (including DVT)
	261	Thrombotic disorders
	219	Pulmonar embolus

Supplementary Table 11: Missing outcome data in matched cohorts.

Outcome	Infected		Uninfected		Vaccinated		Unvaccinated	
	Total	Number (%) with missing data	Total	Number (%) with missing data	Total	Number (%) with missing data	Total	Number (%) with missing data
Small for Gestational Age	4,057	110 (2.7%)	12,171	318 (2.6%)	11,388	387 (3.4%)	22,676	660 (2.9%)
Apgar Score	3,786	123 (3.2%)	11,358	410 (3.6%)	10,752	466 (4.3%)	21,504	848 (3.9%)
Provider-initiated preterm birth AND spontaneous preterm birth	3,603	6 (0.2%)	10,809	4 (0.04%)	11,202	9 (0.1%)	22,404	24 (0.1%)
Provider-initiated very preterm birth AND spontaneous very preterm birth	2,842	1 (0.04%)	8,526	1 (0.01%)	10,057	5 (0.05%)	20,114	3 (0.02%)

Completed and ongoing **pregnancies** [neonates] from Jan 01, 2015 to July 13, 2022
N=575,272 [581,370]

Excluded pregnancies [neonates]*
Completed pregnancies with unknown outcome=**5,737 [5,738]**
Pregnancies ending before 20 weeks gestation, terminations and ongoing pregnancies=**192,620** [**192,724**]
Pregnancies conceived ≥June 2, 2021 [could not reach 40+6 weeks gestation by February 28, 2022]=**16,941** [**17,241**]
Pre-pandemic period pregnancies ending <1 March 2020=**267,532 [271,840]**
Pregnancies completed by May 18, 2020**= 9,781 [9,931]**
Multiple pregnancies**= 1,220 [2,455]**

Pandemic widespread testing period singleton neonates/pregnancies
N=81,441

**Cohort name** ***(outcomes)***	**Cohort exclusion criteria (number excluded)**	**Final matched cohort numbers****	**Final analysis numbers for matched cohorts with missing outcome data*****
**Cohort 1**<br>*Stillbirth*<br>*Extended perinatal mortality*	Neonates of mothers vaccinated in exposure period (N=12,808)†	Infected=4,074<br>Uninfected pool=64,559<br>*Uninfected controls=12,222*	-
**Cohort 2**<br>*Neonatal death*	Neonates of mothers vaccinated in exposure period (N=12,808)<br>Stillbirths (N=221)	Infected=4,060<br>Uninfected pool=64,352<br>*Uninfected controls=12,180*	-
**Cohort 3**<br>*Small for gestational age*<br>*Very small for gestational age*	Neonates of mothers vaccinated in exposure period (N=12,808)<br>Stillbirths (N=221)<br>Neonates<23 or >42 weeks gestation (N=46)	Infected=4,057<br>Uninfected pool=64,309<br>*Uninfected controls=12,171*	Infected=3,947<br>(110 missing data)<br>*Uninfected controls=11,532*<br>*(639 missing data or controls of infected neonates missing data)*
**Cohort 4**<br>*Low Apgar score*<br>*Very low Apgar score*	Neonates of mothers vaccinated in exposure period (N=12,808)<br>Stillbirths (N=221)<br>Neonates<37 weeks gestation (N=4,142)	Infected=3,786<br>Uninfected pool=60,484<br>*Uninfected controls=11,358*	Infected=3,663<br>(123 missing data)<br>*Uninfected controls =10,594*<br>*(764 missing data or controls of infected neonates missing data)*
**Cohort 5**<br>*Any preterm birth*<br>*Spontaneous preterm birth*<br>*Provider-initiated preterm birth*	Neonates≥37 weeks gestation at start of widespread testing (N=2,163)<br>Neonates of mothers vaccinated in exposure period (N=12,424)<br>Stillbirths (N=216)	Infected=3,603<br>Uninfected pool=63,035<br>*Uninfected controls=10,809*	Infected=3,597<br>(6 missing delivery onset)****<br>*Uninfected controls=10,787*<br>*(22 missing delivery onset or controls of infected neonates missing data)*****
**Cohort 6**<br>*Very preterm birth*<br>*Spontaneous very preterm birth*<br>*Provider-initiated very preterm birth*	Neonates≥32 weeks gestation at start of widespread testing (N=6,580)<br>Neonates of mothers vaccinated in exposure period (N=10,855)<br>Stillbirths (N=213)	Infected=2,842<br>Uninfected pool=60,951<br>*Uninfected controls=8,526*	Infected=2,481<br>(1 missing delivery onset)****<br>*Uninfected controls=8,522*<br>*(4 missing delivery onset or controls of infected neonates missing data)*****
**Cohort 7**<br>*Critical care admission or death*	Pregnancies where mother was vaccinated in exposure period (N=12,808)	Infected=4,074<br>Uninfected pool=64,559<br>*Uninfected controls=12,222*	-
**Cohort 8**<br>*Venous thromboembolism*	Pregnancies where mother was vaccinated in exposure period (N=12,808)<br>Pregnancies with venous thromboembolism diagnosis before first infection date in exposure period (N=2)	Infected=4,072<br>Uninfected pool=64,559<br>*Uninfected controls=12,216******	-
**Cohort 9**<br>*Hypertensive disorders of pregnancy*	Pregnancies where mother was vaccinated in exposure period (N=12,808)<br>Pregnancies with hypertensive disorder of pregnancy diagnosis before first infection date in exposure period (N=3)	Infected=4,071<br>Uninfected pool=64,559<br>*Uninfected controls=12,213******	-
**Cohort 10**<br>*Pregnancy-related bleeding*	Pregnancies where mother was vaccinated in exposure period (N=12,808)<br>Pregnancies with pregnancy-related bleeding diagnosis before first infection date in exposure period (N=101)	Infected=3,973<br>Uninfected pool=64,559<br>*Uninfected controls=11,919******	-

Supplementary Figure 1: Selection of pregnancies for the analysis of association between SARS-CoV-2 infection and all neonatal and maternal outcomes.

†This includes 1,429 pregnancies where the woman had both infection and vaccination in the pregnancy exposure period, and 11,379 where the woman had vaccination only.

*Multiple exclusion criteria may have applied for a given pregnancy, but each was only counted in the first exclusion criteria in the order of appearance in this box.
**Each cohort was drawn independently, so the controls will vary between the different cohorts.
***After cohort matching, we removed any of the infected group with missing data for a specific outcome and their controls, as well as any additional controls with missing data.
****Data only missing for delivery onset, so affecting only analyses looking at spontaneous or provider-initiated (very) preterm birth. No missing data for any (very) preterm birth.
*****Exposed pregnancies could only be matched to uninfected control pregnancies that had not had the outcome prior to the gestational week at matching.

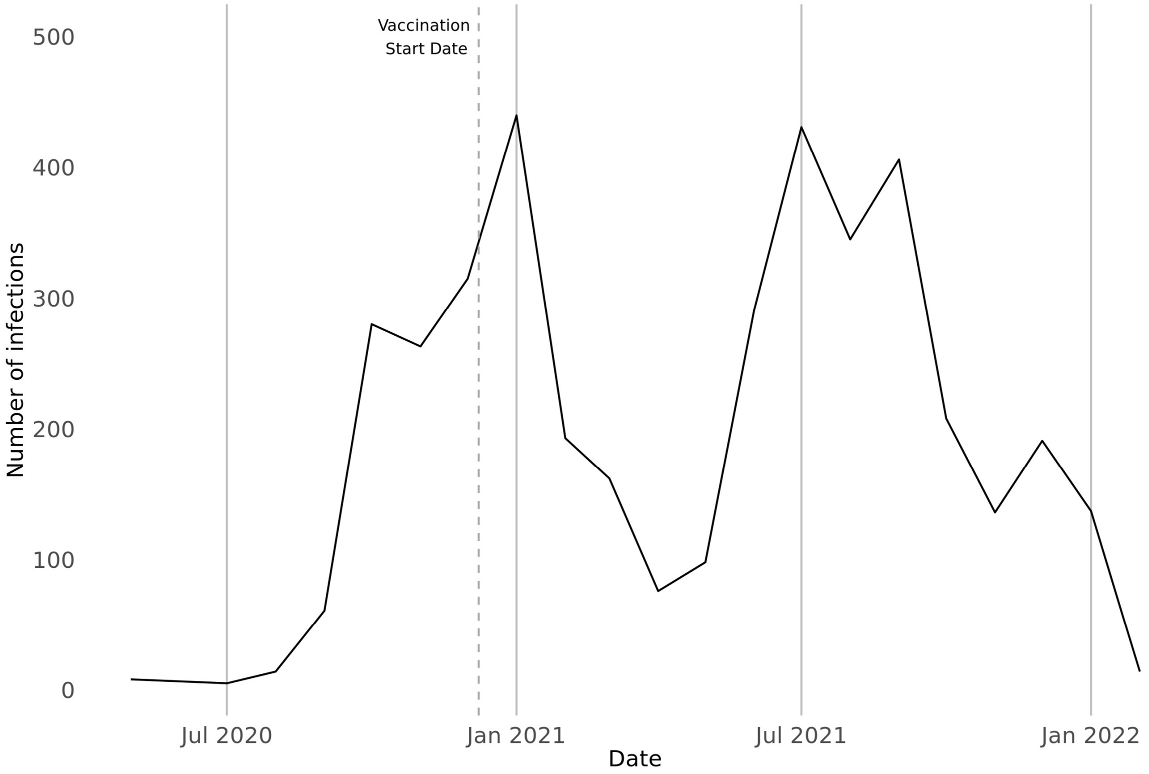

Supplementary Figure 2: Distribution of infections over time in our infected cohort (numbers from cohort 1).

Completed and ongoing **pregnancies** [neonates] from Jan 01, 2015 to July 13, 2022
N=575,272 [581,370]

Excluded pregnancies [neonates]*
Completed pregnancies with unknown outcome=**5,737 [5,738]**
Pregnancies ending before 20 weeks gestation, terminations and ongoing pregnancies=**192,620 [192,724]**
Pregnancies conceived ≥June 2, 2021 [could not reach 40+6 weeks gestation by February 28, 2022]=**16,941 [17,241]**
Pre-pandemic period pregnancies ending <1 March 2020=**267,532 [271,840]**
Pregnancies completed by December 08, 2020= **36,485 [37,070]**
Multiple pregnancies= **790 [1,590]**

Pandemic vaccination period singleton neonates/pregnancies
N=55,167

**Cohort name *(outcomes)***	**Cohort exclusion criteria (number excluded)**	**Final matched cohort numbers****	**Final analysis numbers for matched cohorts with missing outcome data*****
**Cohort 1**<br>*Stillbirth*<br>*Extended perinatal mortality*	Neonates of mothers infected in exposure period (N=5,434)†	Vaccinated=11,379<br>Unvaccinated pool=38,354<br>*Unvaccinated controls=22,758*	-
**Cohort 2**<br>*Neonatal death*	Neonates of mothers infected in exposure period (N=5,434)<br>Stillbirths (N=139)	Vaccinated=11,350<br>Unvaccinated pool=38,244<br>*Unvaccinated controls=22,700*	-
**Cohort 3**<br>*Small for gestational age*<br>*Very small for gestational age*	Neonates of mothers infected in exposure period (N=5,434)<br>Stillbirths (N=139)<br>Neonates<23 or >42 weeks gestation (N=42)	Vaccinated=11,338<br>Unvaccinated pool=38,214<br>*Unvaccinated controls=22,676*	Vaccinated=10,951 (387 missing data)<br>*Unvaccinated controls=21,267 (1,409 missing data or controls of vaccinated neonates missing data)*
**Cohort 4**<br>*Low Apgar score at term*<br>*Very low Apgar score at term*	Neonates of mothers infected in exposure period (N=5,434)<br>Stillbirths (N=139)<br>Neonates<37 weeks gestation (N=2,870)	Vaccinated=10,752<br>Unvaccinated pool=35,972<br>*Unvaccinated controls=21,504*	Vaccinated=10,286 (466 missing data)<br>*Unvaccinated controls=19,761 (1,743 missing data or controls of vaccinated neonates missing data)*
**Cohort 5**<br>*Any preterm birth*<br>*Spontaneous preterm birth*<br>*Provider-initiated preterm birth*	Neonates≥37 weeks gestation at start of vaccination programme (N=1,781)<br>Neonates of mothers infected in exposure period (N=4,718)<br>Stillbirths (N=134)	Vaccinated=11,202<br>Unvaccinated pool=37,332<br>*Unvaccinated controls=22,404*	Vaccinated=11,193 (9 missing delivery onset)****<br>*Unvaccinated controls=22,362 (42 missing delivery onset or controls of vaccinated neonates missing data)*****
**Cohort 6**<br>*Very preterm birth*<br>*Spontaneous very preterm birth*<br>*Provider-initiated very preterm birth*	Neonates≥32 weeks gestation at start of vaccination programme (N=5,730)<br>Neonates of mothers infected in exposure period (N=3,529)<br>Stillbirths (N=134)	Vaccinated=10,057<br>Unvaccinated pool=35,717<br>*Unvaccinated controls=20,114*	Vaccinated=10,052 (5 missing delivery onset)****<br>*Unvaccinated controls=20101 (13 missing delivery onset or controls of vaccinated neonates missing data)*****
**Cohort 7**<br>*Critical care admission or death*	Pregnancies where mother was infected in exposure period (N=5,434)	Vaccinated=11,379<br>Unvaccinated pool=38,354<br>*Unvaccinated controls=22,758*	-
**Cohort 8**<br>*Venous thromboembolism*	Pregnancies where mother was infected in exposure period (N=5,434)<br>Pregnancies with venous thromboembolism diagnosis before first vaccination date in exposure period (N=4)	Vaccinated=11,375<br>Unvaccinated pool=38,354<br>*Unvaccinated controls=34,125******	-
**Cohort 9**<br>*Hypertensive disorders of pregnancy*	Pregnancies where mother was infected in exposure period (N=5,434)<br>Pregnancies with hypertensive disorder of pregnancy diagnosis before first vaccination date in exposure period (N=6)	Vaccinated=11,373<br>Unvaccinated pool=38,354<br>*Unvaccinated controls=34,119******	-
**Cohort 10**<br>*Pregnancy-related bleeding*	Pregnancies where mother was infected in exposure period (N=5,434)<br>Pregnancies with pregnancy-related bleeding diagnosis before first vaccination date in exposure period (N=149)	Vaccinated=11,230<br>Unvaccinated pool=38,354<br>*Unvaccinated controls=22,460******	-

Supplementary Figure 3: Selection of pregnancies for the analysis of association between COVID-19 vaccination and all neonatal and maternal outcomes.

†This includes 1,429 pregnancies where the woman had both vaccination and infection in the pregnancy exposure period, and 4,005 where the woman had infection only.

*Multiple exclusion criteria may have applied for a given pregnancy, but each was only counted in the first exclusion criteria in the order of appearance in this box.
**Each cohort was drawn independently, so the controls vary between the different cohorts.
***After cohort matching, we removed any of the vaccinated group with missing data for a specific outcome and their controls, as well as any additional controls with missing data.
****Data only missing for delivery onset, so affecting only analyses looking at spontaneous or provider-initiated (very) preterm birth. No missing data for any (very) preterm birth.
*****Exposed pregnancies could only be matched to unvaccinated control pregnancies that had not had the outcome prior to the gestational week at matching.

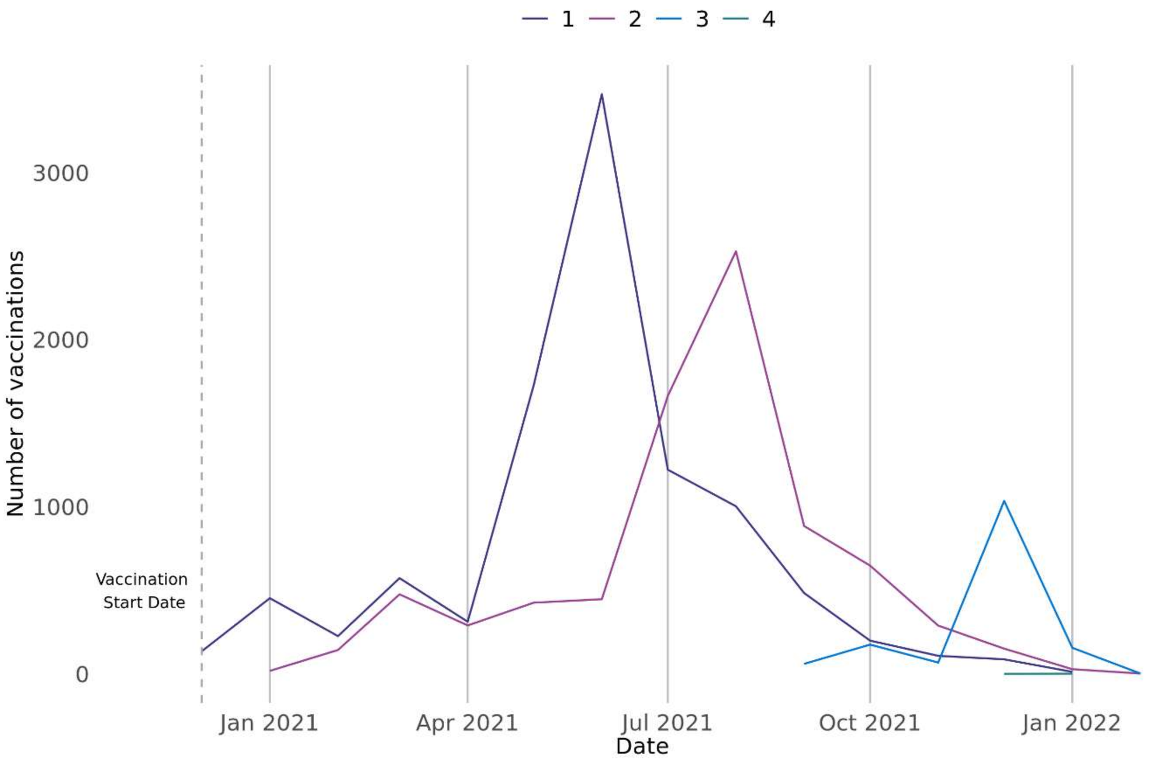

Supplementary Figure 4: Distribution of vaccinations over time in our vaccinated cohort (numbers from cohort 1), by vaccine dose number.

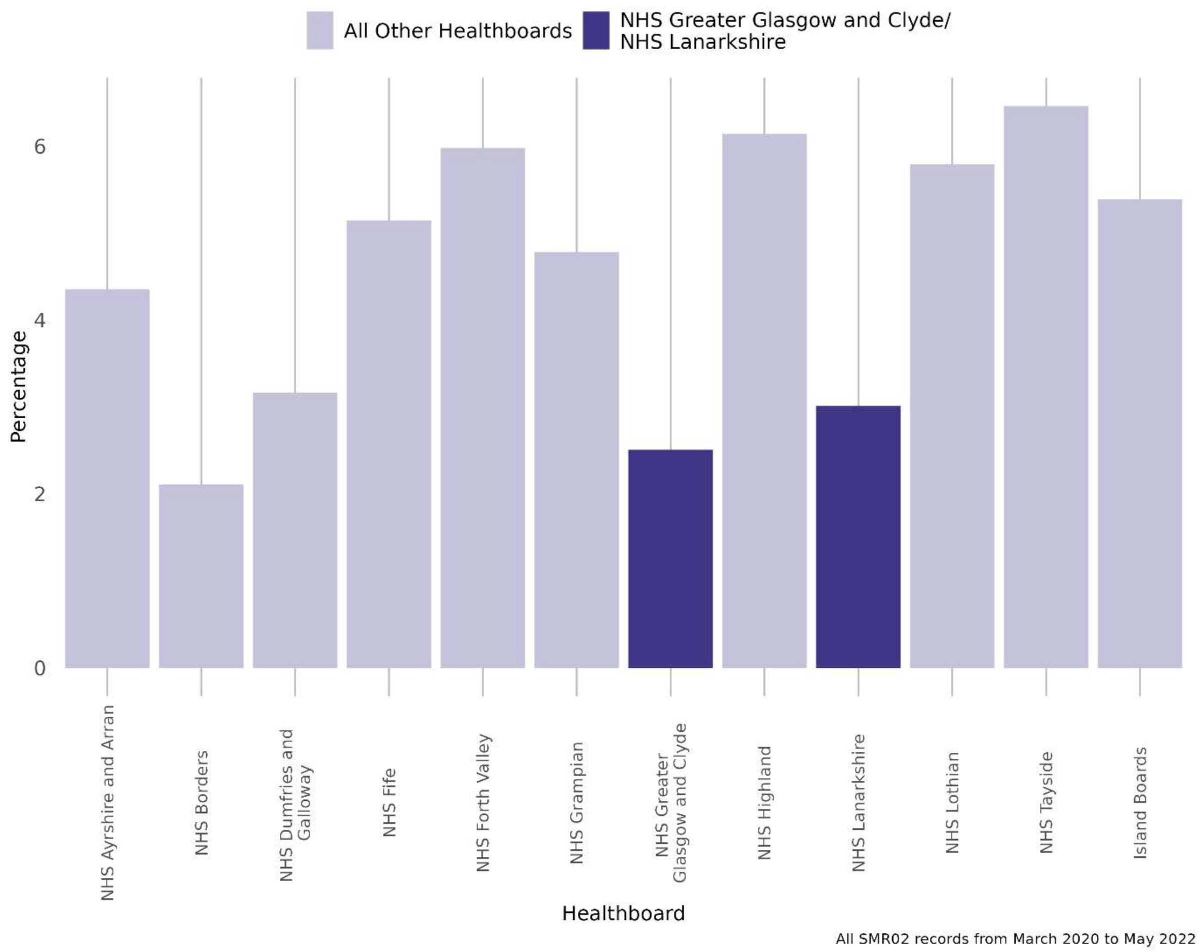

Supplementary Figure 5: Percentage of all maternity records between March 2020 and May 2022 with hypertensive disorders of pregnancy coded as a diagnosis by healthboard.

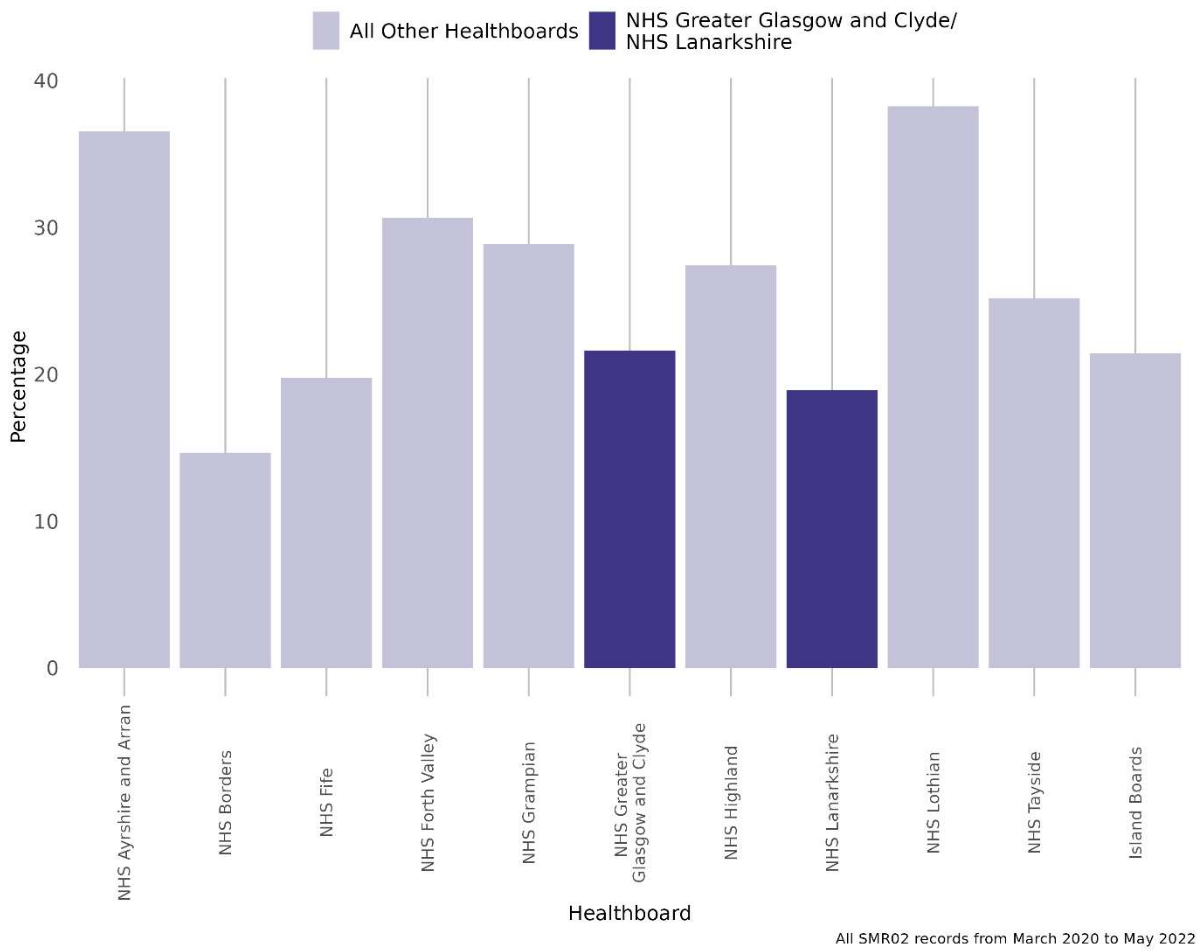

Supplementary Figure 6: Percentage of all maternity records between March 2020 and May 2022 with pregnancy-related bleeding coded as a diagnosis by healthboard.
