# Supplementary material for: Neonatal and maternal outcomes following SARS-CoV-2 infection and COVID-19 vaccination: a population-based matched cohort study
Source: Nat Commun. 2023 Aug 29;14:5275. doi: 10.1038/s41467-023-40965-9 (PMC10465539; doi:10.1038/s41467-023-40965-9)
Supplement: Supplementary file 3 — Reporting Summary [file 41467_2023_40965_MOESM3_ESM.pdf]

nature portfolio

Corresponding author(s): Rachael Wood

Last updated by author(s): Jul 28, 2023

Reporting Summary

Nature Portfolio wishes to improve the reproducibility of the work that we publish. This form provides structure for consistency and transparency in reporting. For further information on Nature Portfolio policies, see our Editorial Policies and the Editorial Policy Checklist.

Statistics

For all statistical analyses, confirm that the following items are present in the figure legend, table legend, main text, or Methods section.

n/a	Confirmed	
☐	☒	The exact sample size (*n*) for each experimental group/condition, given as a discrete number and unit of measurement
☐	☒	A statement on whether measurements were taken from distinct samples or whether the same sample was measured repeatedly
☐	☒	The statistical test(s) used AND whether they are one- or two-sided<br>*Only common tests should be described solely by name; describe more complex techniques in the Methods section.*
☐	☒	A description of all covariates tested
☐	☒	A description of any assumptions or corrections, such as tests of normality and adjustment for multiple comparisons
☐	☒	A full description of the statistical parameters including central tendency (e.g. means) or other basic estimates (e.g. regression coefficient) AND variation (e.g. standard deviation) or associated estimates of uncertainty (e.g. confidence intervals)
☐	☒	For null hypothesis testing, the test statistic (e.g. *F*, *t*, *r*) with confidence intervals, effect sizes, degrees of freedom and *P* value noted<br>*Give P values as exact values whenever suitable.*
☒	☐	For Bayesian analysis, information on the choice of priors and Markov chain Monte Carlo settings
☒	☐	For hierarchical and complex designs, identification of the appropriate level for tests and full reporting of outcomes
☒	☐	Estimates of effect sizes (e.g. Cohen's *d*, Pearson's *r*), indicating how they were calculated

Our web collection on statistics for biologists contains articles on many of the points above.

Software and code

Policy information about availability of computer code

Data collection	R version 3.6.1
Data analysis	R version 3.6.1

For manuscripts utilizing custom algorithms or software that are central to the research but not yet described in published literature, software must be made available to editors and reviewers. We strongly encourage code deposition in a community repository (e.g. GitHub). See the Nature Portfolio guidelines for submitting code & software for further information.

Data

Policy information about availability of data

All manuscripts must include a data availability statement. This statement should provide the following information, where applicable:

- Accession codes, unique identifiers, or web links for publicly available datasets
- A description of any restrictions on data availability
- For clinical datasets or third party data, please ensure that the statement adheres to our policy

Aggregate data files on COVID-19 vaccinations and SARS-CoV-2 infections among pregnant women are available here: https://www.opendata.nhs.scot/organization/health_protection. Patient-level data underlying this article cannot be shared publicly due to data protection and confidentiality requirements. Public Health Scotland is the data holder for the data used in this study. Data can be made available to approved researchers for analysis after securing relevant permissions from the data holders via the Public Benefit and Privacy Panel. Enquiries regarding data availability should be directed to phs.edris@phs.scot.

Research involving human participants, their data, or biological material

Policy information about studies with human participants or human data. See also policy information about sex, gender (identity/presentation), and sexual orientation and race, ethnicity and racism.

Reporting on sex and gender	We refer to women/mothers throughout the paper when discussing the exposure in pregnancy and maternal socio-demographic and clinical characteristics. No data are presented on sex of babies as this is not relevant to our study question.
Reporting on race, ethnicity, or other socially relevant groupings	Ethnicity is reported as a covariate of interest in this study. We have categorized Ethnicity into five groups (White/South Asian/ Black or Caribbean or African/Other or Mixed/Unknown) according to the Scottish decennial population census groups (https://www.ndc.scot.nhs.uk/Dictionary-A-Z/Definitions/index.asp?Search=E&ID=243&Title=Ethnic%20Group).
Population characteristics	Singleton neonates/pregnancies that reached at least 20 weeks gestation and ended in a live birth or stillbirth. Firstly, we selected all neonates exposed to SARS-CoV-2 infection (but not COVID-19 vaccination) between six weeks preconception and the end of pregnancy as well as three control neonates for each infected neonate who were not exposed to either maternal COVID-19 vaccination or SARS-CoV-2 infection in the same pregnancy risk period matched on maternal age at conception, season of conception and gestational age at time of infection/matching. Secondly, we selected all neonates exposed to maternal COVID-19 vaccination (but not SARS-CoV-2 infection) between six weeks preconception and the end of pregnancy as well as two control neonates for each vaccinated neonate who were not exposed to either maternal COVID-19 vaccination or SARS-CoV-2 infection in the same pregnancy risk period matched on maternal age at conception and gestational age at time of vaccination/matching. We provide detailed descriptive information on these cohorts in Table 2 in the paper.
Recruitment	National, prospective dynamic cohort of routinely collected healthcare data.
Ethics oversight	The National Research Ethics Service Committee, South East Scotland 02 provided ethical approval for COPS (REC 12/SS/0201: SA 2). The Public Benefit and Privacy Panel for Health and Social Care provided information governance approval (2021-0116).

Note that full information on the approval of the study protocol must also be provided in the manuscript.

Field-specific reporting

Please select the one below that is the best fit for your research. If you are not sure, read the appropriate sections before making your selection.

☒ Life sciences ☐ Behavioural & social sciences ☐ Ecological, evolutionary & environmental sciences

For a reference copy of the document with all sections, see nature.com/documents/nr-reporting-summary-flat.pdf

Life sciences study design

All studies must disclose on these points even when the disclosure is negative.

Sample size	Sample size calculations were conducted and are presented in the protocol. We ultimately included all eligible neonates in our exposed groups based on this national level dataset, with appropriate numbers of unexposed controls selected.
Data exclusions	Data were excluded if there were pre-specified unfeasible values, as described in the data dictionary available from: https://github.com/Public-Health-Scotland/COPS-public.
Replication	All code was double checked by a second analyst to ensure it was correct.
Randomization	This was an observational study so we did not conduct randomization. We controlled for confounders by matching our exposed groups of neonates to unexposed groups of neonates (1:3 matching or 1:2 matching depending on exposure) by key confounders (e.g., maternal at at conception), with other confounders adjusted for in the conditional logistic regression model (e.g, area-level deprivation and clinical vulnerability).
Blinding	Analysts preparing the dataset from the routine health records were not formally blinded to the exposure status of the neonates in the analysis. The exposure and outcome data were drawn from different data sources, however, so in practice the analysts were unaware of the exposure status when extracting and preparing the outcome data and vice versa. Due to the use of routine data which require careful checking before running the models (e.g., knowing the distribution of different covariates and levels of missing data by exposure status), the analysts conducting all the analyses were not blinded to the exposure groups. To minimize the potential for any bias introduced for lack of blinding, we prepared a detailed protocol and analysis plan that was followed throughout the analysis.

Reporting for specific materials, systems and methods

We require information from authors about some types of materials, experimental systems and methods used in many studies. Here, indicate whether each material, system or method listed is relevant to your study. If you are not sure if a list item applies to your research, read the appropriate section before selecting a response.

Materials & experimental systems

n/a	Involved in the study
☒	☐ Antibodies
☒	☐ Eukaryotic cell lines
☒	☐ Palaeontology and archaeology
☒	☐ Animals and other organisms
☒	☐ Clinical data
☒	☐ Dual use research of concern
☒	☐ Plants

Methods

n/a	Involved in the study
☒	☐ ChIP-seq
☒	☐ Flow cytometry
☒	☐ MRI-based neuroimaging
